# Disappearance of soft drusen and subsequent development of choroidal neovascularization following macular hole surgery: a case report

**DOI:** 10.1186/s12886-015-0029-8

**Published:** 2015-04-28

**Authors:** Ji Hwan Lee, Taekjune Lee, Sung Chul Lee, Christopher Seungkyu Lee

**Affiliations:** Department of Ophthalmology, Institute of Vision Research, Yonsei University College of Medicine, 134 Shinchondong, Seodaemungu, Seoul Republic of Korea

**Keywords:** Drusen, Choroidal neovascularization, Macular hole, Surgery

## Abstract

**Background:**

Drusen are important risk factor for neovascular age-related macular degeneration (AMD) and have a dynamic nature as they can enlarge, newly form, or disappear over time. There have been few reports on drusen regression or choroidal neovascularization (CNV) development after macular hole surgery. We report, to our knowledge, the first case of both drusen regression and subsequent CNV development within 7 months of successful macular hole surgery.

**Case presentation:**

A 73-year-old woman presented with a stage 3 full-thickness macular hole and large, confluent soft macular drusen in the right eye and a neovascular age-related macular degeneration (AMD) in the fellow eye. Four months after the successful macular hole surgery, significant regression of drusen was seen, especially in the temporal area to the fovea. Three months later, the patient developed CNV and her best-corrected visual acuity decreased to 20/100, despite further regression of macular drusen.

**Conclusions:**

Macular hole patients with macular soft drusen need to be carefully followed up after surgery for possible drusen regression and CNV development.

## Background

Large, soft drusen are associated with a greater risk for developing advanced age-related macular degeneration (AMD), including choroidal neovascularization (CNV) and geographic atrophy [1]. Drusen have a dynamic nature as they can enlarge, newly form, or disappear over time [2-4]. There have been case reports on drusen disappearance after macular hole surgery [5-7]. Macular hole surgery has also been associated with the development CNV [8-10]. This is the first report to our knowledge in which both phenomena occurred together in the same eye. We report a patient who showed a rapid regression of soft macular drusen and the development of CNV within 7 months of successful macular hole surgery.

## Case presentation

**Figure 1:**
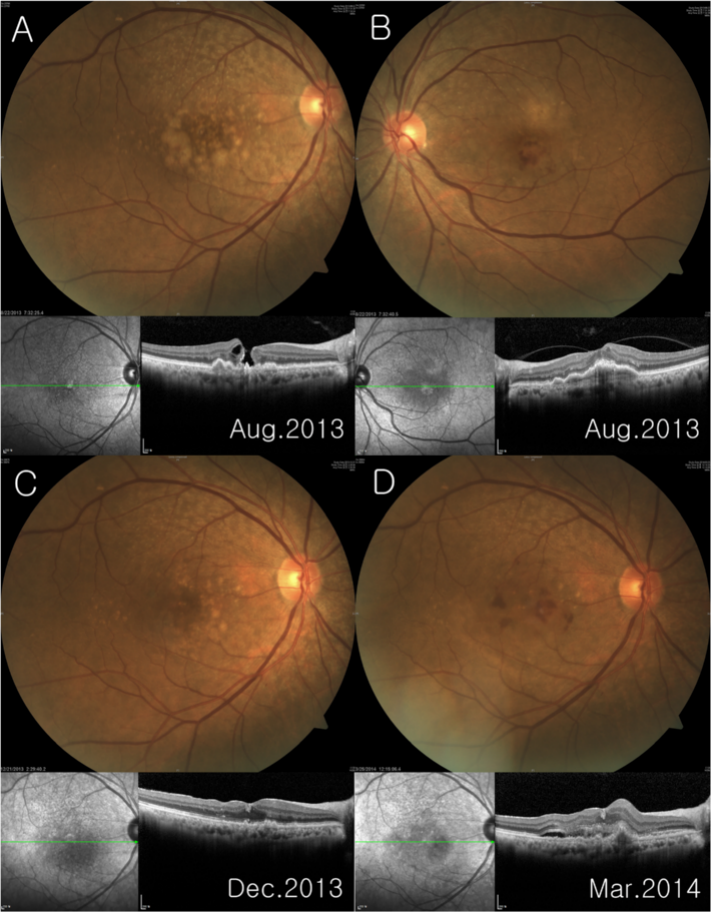
Color fundus photography and corresponding spectral-domain optical coherence tomography (OCT) scan through the fovea **A** Stage 3 full-thickness macular hole with large, soft, confluent macular drusen was noted in the right eye **B** The left eye fundus and OCT scan show a neovascular age-related macular degeneration with hemorrhage in the macula, for which she had undergone four sessions of intravitreal ranibizumab injections. Note the vitreomacular adhesion at the foveal center **C** Macular hole was closed successfully and preexisted macular drusen, especially in temporal area to the fovea, significantly regressed at 4 months after macular hole surgery in the right eye **D** Three months later, further regression of macular drusen was noted, but choroidal neovascularization with subretinal fluid and hemorrhage developed.

A 73-year-old woman presented with decreased visual acuities in her both eyes. The previous medical history was unremarkable except for hypertension. Ophthalmological examinations including funduscopy, fluorescein/indocyanine green angiography and spectral-domain optical coherence tomography (OCT) showed a stage 3 full-thickness macular hole and large, confluent soft drusen in the right eye and a neovascular AMD in the left eye, for which she had undergone four sessions of intravitreal ranibizumab injections in other hospital (Figure 1A,B). Her best-corrected visual acuity (BCVA) was 20/63 in the right eye and 20/50 in the left eye. She underwent an additional intravitreal ranibizumab injection in the left eye and the macular hole surgery including vitrectomy, internal limiting membrane (ILM) peeling using indocyanine green dye, and 17% SF_6_ gas tamponade in the right eye. The fluid at the base of macular hole was not aspirated during fluid-air exchange to avoid damage to underlying retinal pigment epithelium (RPE). After a week of prone positioning, OCT showed a closed macular hole. Four months after the surgery, fundus showed drusen regression, especially in the temporal area to the fovea, and the BCVA improved to 20/50 (Figure 1C). However, 3 months later, the patient developed CNV and her BCVA decreased to 20/100, despite further regression of macular drusen (Figure 1D). Her fellow eye CNV also recurred and she was treated with additional intravitreal ranibizumab injections in both eyes.

### Discussion

This case illustrated the regression soft macular drusen and subsequent development of CNV after successful macular hole surgery, which may have acted as an exogenous stimuli for the regression of drusen and the development of advanced AMD. Spontaneous regression of drusen is well documented, but drusen regression does not necessarily indicate beneficial consequence since the development of CNV or geographic atrophy can ensue [11]. It only took 3 months to develop CNV after drusen regression in the present case. Although it may be possible that drusen regression occurred spontaneously irrespective of surgery, relatively short period between surgical intervention and the onset of drusen disappearance suggest possible causal relationship. There have been three case reports on the drusen regression following macular hole surgery [5-7]. Their association, however, is not clear as one study showed that preoperative macular drusen in three patients with macular holes remained unchanged following surgery [12].

The mechanism of drusen disappearance after macular hole surgery is uncertain. Holz et al. proposed several possibilities including phagocytosis of drusen material by macrophages or multinucleated giant cells, phagocytosis by newly proliferated RPE, and reactive RPE changes to exogenous stimuli resulting in phagocytosis of drusen material [5]. This seems plausible since multinucleated giant cells are in intimate association with Bruch’s membrane as their cytoplasmic extensions are on Bruch’s membrane and at the margin of Bruch’s membrane defects [13].

In the present case, vitrectomy, ILM peeling, and gas tamponade were performed to close macular hole. ILM peeling may not be a significant factor associated with drusen regression; Holz et al. did not attempt to peel ILM during his macular hole surgery, whereas Dithmar et al. did, but both noted the disappearance of drusen after surgery [5,6]. Other surgical components such as induction of posterior hyaloid detachment, vitrectomy, and gas tamponade may somehow acted as exogenous stimuli to the exposed RPE and perhaps accelerated phagocytosis of multinucleated giant cells, resulting in degradation of drusen material around the macular hole.

There have been few case reports on CNV development after macular hole surgery [8-10]. Age-related degenerative changes in Bruch’s membrane and RPE are regarded as the major underlying factor for the development of CNV [9]. Macular hole surgery may have caused the degenerated Bruch’s membrane to further breakdown by mechanical effects and/or activation of multinucleated giant cells, providing an angiogenic stimulus for CNV [13]. However, it should be noted that our patient may have been prone to develop CNV since CNV had already developed in the fellow eye. The unoperated fellow eye with CNV is complicated with a posterior vitreomacular adhesion (Figure 1B) that has been regarded as the risk factor of neovascular AMD [14]. It is interesting that the eye with macular hole still developed neovascular CNV after posterior hyaloid was removed during vitrectomy.

## Conclusion

Careful follow-up is warranted in macular hole patients with soft drusen since drusen regression and CNV development may occur after macular hole surgery.

## Consent

Written informed consent was obtained from the patient for publication of this case report and any accompanying images. A copy of the written consent is available for review by the Editor of this journal.

## Abbreviations

AMD: Age-related macular degeneration
CNV: Choroidal neovascularization
OCT: Optical coherence tomography
BCVA: Best-corrected visual acuity
ILM: Internal limiting membrane
RPE: Retinal pigment epithelium

## References

[1] Holz FG, Wolfensberger TJ, Piguet B, Gross-Jendroska M, Wells JA, Minassian DC (1994). Bilateral macular drusen in age-related macular degeneration. Prognosis and risk factors. Ophthalmology.

[2] Sarks SH (1980). Drusen and their relationship to senile macular degeneration. Aust J Ophthalmol.

[3] Sebag M, Peli E, Lahav M (1991). Image analysis of changes in drusen area. Acta Ophthalmol.

[4] Bressler NM, Munoz B, Maguire MG, Vitale SE, Schein OD, Taylor HR (1995). Five-year incidence and disappearance of drusen and retinal pigment epithelial abnormalities. Arch Ophthalmol.

[5] Holz FG, Staudt S (2001). Disappearance of soft drusen following macular hole surgery. Retina.

[6] Dithmar S, Pollithy S, Ach T (2013). Disappearance of central confluent soft drusen following vitrectomy and ILM peeling. Eye.

[7] Lehmann F, Jenisch T, Helbig H, Gamulescu M. Drusen characteristics after internal limiting membrane peeling. Ophthalmologe. 2014 (in press). 10.1007/s00347-013-3007-7 24549686

[8] Banker A, Freeman W, Kim J, Munguia D, Azen S (1997). Vision-threatening complications of surgery for full-thickness macular holes. Vitrectomy for macular hole study group. Ophthalmology.

[9] Tabandeh H, Smiddy WE, Sullivan PM, Monshizadeh R, Rafiei N, Cheng L (2004). Characteristics and outcomes of choroidal neovascularization occuring after macular hole surgery. Retina.

[10] Oh HN, Lee JE, Kim HW, Yang JW, Yun IH (2012). Occult choroidal neovascularization after successful macular hole surgery treated with ranibizumab. Clin Ophthalmol.

[11] Yehoshua Z, Wang F, Rosenfeld PJ, Penha FM, Feuer WJ, Gregori G (2011). Natural history of drusen morphology in age-related macular degeneration using spectral domain optical coherence tomography. Ophthalmology.

[12] Chaudhry NA, Flynn HWJ, Smiddy WE, Thompson JT (2000). Macular hole surgery in the presence of prominent macular drusen. Arch Ophthalmol.

[13] Dastgheib K, Green R (1994). Granulomatous reaction to Bruch’s membrane in age-related macular degeneration. Arch Ophthalmol.

[14] Lee SJ, Lee CS, Koh HJ (2009). Posterior vitreomacular adhesion and risk of exudative age-related macular degeneration: paired eye study. Am J Ophthalmol.

